# Improving the therapist’s metacognition and capacity to intersubjectively attune with a patient with psychosis through the exploration of the therapist’s developmental history: a case report

**DOI:** 10.3389/fpsyt.2023.1195695

**Published:** 2023-06-26

**Authors:** Gianpaolo Salvatore, Tania Di Somma, Luisa Buonocore, Maria Conza, Nadia Di Sturco, Gerardina Fimiani, Nicoletta Manfredi, Raffaella Marciano, Antonella Pallotta, Maria Grazia Proto, Anna Sateriale

**Affiliations:** ^1^Department of Humanities, Letters, Cultural Heritage, Education Sciences, University of Foggia, Foggia, Italy; ^2^Studio Maya, Psychiatry, Psychotherapy, Criminology, Research, Training, Salerno, Italy

**Keywords:** psychosis, developmental trauma, clinical supervision, intersubjective attunement, therapeutic relationship, metacognition, mentalization

## Abstract

Clinical literature emphasizes how symptoms of psychosis can be efficiently targeted by psychological treatments. The most well-known approach to these symptoms is cognitive-behavioral therapy; but in the last few decades also other approaches are enriching the landscape, focusing on the dysfunctions in mentalization or metacognition, a spectrum of mental activities involving thinking about one’s own and others’ mental states. This huge amount of theoretical reflection and empirical research focused on the implementation of treatments does not seem to be associated with an attention to the inner world of the therapist who relates to the patient with psychosis; for example, to the impact of the therapist’s developmental history on the therapeutic relationship. In this paper the authors are inspired by an intersubjective perspective, according to which although the treatment is for the patient’s benefit, both the patient’s and the therapist’s developmental history and psychological organization are equally relevant for understanding the clinical exchange. On this basis, the authors make a “parallel” analysis of the clinical case of a young woman with symptoms of psychosis (i.e., persecutory delusions, auditory verbal hallucinations, social withdrawal) and its supervision process. They show how the therapeutic relationship can be significantly conditioned by the therapist’s developmental history; and how a process of supervision focused on the exploration of the traumatic elements of this history can effectively promote the therapist’s metacognitive capabilities, a functional patient-therapist intersubjective attunement, and a good clinical outcome.

## 1. Introduction

Although effective pharmacological interventions for patients with psychosis have been developed in the last few decades, 20%–40% of patients are drug-resistant or residually symptomatic in the long-term treatment (1), or experience side effects like weight gain, which is associated with low self-esteem, social isolation, medication non-compliance (2), and an alteration of the body self-image that can exacerbate symptoms (3). This has increased the need for efficient psychotherapeutic approaches to psychosis over the years. The most well-known psychotherapeutic intervention is cognitive behavioral therapy (CBT) (4), primarily focused on helping clients to regulate negative emotions correlated to symptoms, question the cognitions boosting them, and reducing stigma. More recent—“third wave”—CBT focuses on symptom sustaining factors, such as repetitive thinking or intolerance of uncertainty (5) and cognitive biases (6). Many people with psychosis, however, do not respond positively to these approaches predominantly focused on symptoms, and end treatment with significant residual symptomatology (7). This has contributed to give impetus to new therapeutic models that focus on the causal factors of symptoms, such as the impairment of patient’s sense of self (8) and—more specifically—dysfunctions in processes through which patients make meaning of their own and others’ mental states, namely mentalization (9) or metacognition^1^ (10, 11). A core tenet of these approaches is the idea that dysfunctions in mentalization/metacognition can be improved in the context of a therapeutic relationship seen as “an intersubjective process occasioned by the meeting of two minds” [(12), p. 87; see also (13)]. In line with some psychoanalytic authors, it is possible to radicalize this intersubjective perspective, postulating that everything happening in the clinical encounter could be considered the expression of an intersubjective field in which each participant brings a relevant contribution (14, 15). From this perspective, the intersubjective process that takes form in the clinical encounter may result not only in an increase in the patient’s mentalization/metacognition capacity, but also in negative fluctuations, which may be associated with similar fluctuations on the part of the therapist; for example, when the patient’s poor metacognitive ability manifests itself in overly concrete or “black and white” thinking, the therapist may tend to show a complementary tendency to argue competitively with the patient and/or impose his or her own view of reality (16).

The radically intersubjective perspective summarized so far implies an overcoming of the medical paradigm whereby a therapist objectively analyzes the patient’s disease, remaining completely extraneous to the object of exploration, and then cures that disease according to a specific protocol; on the contrary, the therapist, with her/his psychological organization, is deeply involved in the events she/he is trying to decipher and deal with: although the treatment is for the patient’s benefit, both the patient’s and the therapist’s developmental story, psychological organization, and processes of making sense of their own and the patient’s experience are equally relevant for understanding the clinical exchange. The application of this perspective to the argumentative context of this paper implies that in order to deeply understand the patient with schizophrenia, the therapist is called to remember that therapist and patient may share some nuclear affective elements; for example, the difficulty to tolerate both proximity and loneliness in relationships with others (17, 18). In this perspective, client and therapist can both be considered wounded human beings involved in a process aimed at helping the client (19). Psychosis seems correlated with traumatic experiences (20); so current psychotherapeutic approaches are enriched with the prospect of fostering patients’ ability to make sense of the mental states (e.g., feeling of being persecuted) correlated to trauma (21). This seems also to concern the other part of the “wounded dyad”: various studies show that developmental trauma (DT) seems particularly present in the therapists’ experiential baggage (22, 23).

Despite this data, there is a general lack of attention to the way the therapist’s traumatic suffering impacts the therapeutic relationship, least of all with a patient with psychosis. This seems also reflected in the limited attention paid to these aspects in the context of clinical supervision in general. Several authors emphasize the role of the supervisory relationship as a kind of attachment relationship (24, 25) that can be internalized in the therapist’s work as a means of self-support and self-review (26–28). However, this kind of support from the supervisor seems conceived as a resource to deal with the technical and transferential issues posed by the patient. Not much emphasis seems to be placed, for example, on how exactly the supervision should tackle therapists’ developmental trauma (DT) and its potential negative impact on the therapeutic relationship and the clinical process.

Based on the above, there is room for improvement of therapeutic interventions inspired by an intersubjective paradigm and focused on dysfunction in metacognition/mentalization, paying due attention to the internal world of the therapist engaged with a person with psychosis. Some authors seem to go in this direction, deepening the theoretical exploration of countertransference processes in the therapy of patients with psychosis [see (12, 29, 30)]. It seems possible, however, to go further, focusing on the therapist’s DT—which is a substantial element underlying countertransference—and its impact on the therapist’s metacognitive capabilities and disposition to intersubjectively attune with patients. This aim seems coherent with what Winnicott (31) observed: “If we are able to be the analysts of psychotic patients, we must have reached down to very primitive things in ourselves” (p. 61); and with what more recently Horowitz (29) observed: “we must reach deep down inside to touch the parts of ourselves that have been wounded, endeavoring to move freely in those aspects of experience that most closely mirror the closed universe of sorrow and loss that envelops many afflicted with schizophrenia” (p. 241).

In this paper, through a clinical case study, we show how the therapist’s psychological organization related to DT predisposes her to a contingent impairment of her metacognitive functions in contingent problematic phases of the therapeutic relationship; and prevents the therapist from intersubjectively attuning with the patient. Moreover, we show how a process of supervision specifically focused on fostering the therapist’s metacognitive ability to make sense of her own traumatic suffering and its impact on the therapeutic relationship can effectively promote a change of the therapist’s emotional disposition toward the patient, unlock the therapist’s ability to intersubjectively attune with the patient’s suffering, guide the therapist’s line of intervention, and most likely contribute to a good clinical outcome.

## 2. The case of Patricia

Patricia was in her early 20s when her parents brought her for therapy. She was an only child, and lived with her parents. Her father was a 56-year-old professional; she described him as severe and judgmental. Her mother was a 50-year-old housewife, described as cold, perfectionist, and controlling. Patricia grew up with a constant fear of mistakes and of disappointing her parents’ expectations. Simultaneously, in line with the standards set by her mother, Patricia displayed perfectionistic tendencies and neglected social relations, dedicating herself body and soul to studying. She was studying for her first exam session at the university when she began to manifest an initial psychotic breakdown, in the form of mental confusion, persecutory delusions and auditory verbal hallucinations. She was convinced that during the lessons the teachers told the whole class, through “a secret communication code,” that Patricia was “unreliable and unable to be in the world,” and she heard whispering voices insulting her. This prompted her to abandon lessons. She gradually lost the ability to concentrate on her studies, and had fallen into severe social withdrawal and apathy. At first, her parents criticized Patricia for neglecting her studies and for the absurdity of her delusional thoughts. When they witnessed the worsening of her condition, they asked for therapeutic help. Patricia started a pharmacological treatment with a public health psychiatrist (Olanzapine, 10 mg). This treatment drastically reduced hallucinations and partially regulated persecutory delusions: even though the patient no longer showed a structured delusion, a recurring doubt that her colleagues and neighbors had malicious intentions toward her persisted. Patricia also started individual psychotherapy, delivered in a private outpatient clinic. Individual sessions were weekly and lasted about 45 min. Patricia attended more than 90% of the scheduled weekly appointments. The psychotherapist, Judy,^2^ was in her early 40s, she had a cognitive behavioral background, a thorough knowledge of the literature on the relationship between metacognition and psychopathology and about 10 years of clinical experience working with persons with severe mental illnesses. During her training, she had undergone 2 years of cognitive-analytic therapy.

## 3. The supervision

Immediately after the end of each therapy session, Judy took written notes on the highlights of the meeting and her own impressions and emotions. During the first 2 months of therapy, she had the overall impression of a cooperative atmosphere in the therapeutic relationship. Then, in a session of the third month, Judy tried to help Patricia to understand that the negative judgment she placed on her professors and colleagues reproduced the severity with which she tended to judge herself. Patricia left the session with a perplexed expression, and after a few hours she texted Judy that she intended to suspend the therapy, because she thought that Judy had badmouthed her to her university professors. Judy called her and struggled to persuade her to discuss the matter in the next session, and Patricia listlessly agreed to return. What happened in the following session made Judy feel confused and prompted her to ask one of the authors for a clinical supervision. The following excerpt is taken from the transcription of the audio-registration of this supervision:

Supervisor (S): can we focus on that specific scene? She is sitting in front of you and you want to discuss what had happened…do you remember what you said, and what happened?

Judy (J): yes… I wanted to reassure her that her suspicions did not correspond to reality…I said something like “Patricia, I’m sorry if I may have somehow led you to think this, but I assure you that I have had no contact with your professors, I could never do this to you. Rather, maybe we could talk about how it makes you feel not being able to have full confidence in me just now”…but it seemed like I was making things worse…she looked at me with a suspicious expression, she did not talk, she hardly answered my questions…

S: …uhm…how did you feel?

J: well, she keeps her head down while I speak…and I feel, you know, kind of…under scrutiny…then when I finish the sentence, she looks at me…(*pause*).

S: how is she looking at you?

J: uhm…with a kind of detached expression…and disapproving, too…

S: uhm…was there this sense of her detachment and disapproval?!

J: yes…

S: please, try to recall in yourself this feeling you had when you faced her detachment…can you?

J: I think so…

S: are you feeling it right now?

J: I think so…well…I think it is unfair, that I am trying so hard and she does not help me to help her…

S: you look angry while you say this…are you feeling angry while Patricia has this expression of disapproving detachment?

J: yes… I guess I think she is ungrateful and unjust to me

(*pause*)

S: I think this is understandable…but…listen, I am trying to put myself in your shoes in front of that gaze of Patricia, and I can experience your own angry sense of injustice … but if I look inside, I see in myself something that precedes this anger, something more painful … that could make me react with anger…I wonder if something similar may have happened to you, too…

J: (*sad expression*).

S: your expression has changed. What are you feeling now while Patricia is looking at you with that gaze?

J: (*pause*)…it’s a sort of weakness…sadness…

S: uhm…is there any image that comes to your mind right now?

(*long pause*)

J: yes…it is very difficult for me to say this…I see my mother’s face looking at me with no expression, it’s empty (moved)… it was an expression that could suddenly change and become angry at me, even if I had done nothing wrong…

In the following part of the supervision, Judy was able to tell the supervisor that growing up she had suffered the consequences of her mother’s severe dysphoric depression, and her father’s physical and emotional detachment. The supervisor helped Judy to see that perhaps her urge to reassure the patient about the purity of her intentions reenacted the child part of Judy’s identity who had tried countless times to “apologize” to her mother for being wrong, in an attempt to get a signal of love from her or to avoid her anger. It was as if in front of the patient’s apparent “detachment” and “disapproval,” Judy was again—at a procedural level—in front of her mother and was saying “please forgive me for not being the daughter you want, a daughter capable of making you happy.” The supervisor also helped Judy to see that, as had happened with her mother, also in the interaction with Patricia the failure of this attempt generated a state of “switching off” (weakness) and psychological pain: a state of psychological collapse, slowing down of vital functions (32). Judy also recognized that this tendency to fear detachment and disapproval had also occurred with other patients. Moreover, she recognized that she had addressed these issues of her developmental history during her personal psychotherapy, but she had never realized how these traumatic contents were implicitly reactivated in interactions with patients. Finally, on this basis Judy and the supervisor came to understand that there was a sort of symmetry in the internal traumatic dynamics of the members of the therapeutic dyad: in Patricia, an identity part guided by suspiciousness (at times delusional), detachment and withdrawal from the relationship constituted a sort of strategy to protect a traumatized and painful identity part; similarly, in Judy, a relational strategy based on “apologizing” and trying to conform to how she imagined the other wanted her to be, protected a traumatized part related to the repeated experience of her mother’s detachment and anger.^3^

At the end of the supervision, Judy told the supervisor that she felt relieved and that she could really “see” Patricia’s suffering now.

## 4. The impact of supervision on therapy

The therapeutic session following the supervision started with a long silence. Although Patricia had a diffident expression, Judy felt serene, and she felt no urgency to make any intervention. Thanks to the supervision, she was aware that the experience of feeling understood about her DT and becoming able to attune with Patricia’s own DT could be considered the two sides of the same coin. It was this awareness that prompted her to make the following intervention: “Listen, Patricia, I want to share with you what is going on inside of me right now. There is a part of me that feels in trouble because she feels like she is walking on eggshells out of fear that any of my words might hurt, offend or harm you, or, maybe, scare you; but then there is also another part that manages to be in touch with a need in Patricia, which is not clearly focused, which brings her here in front of me, despite the risks that this relationship may entail.” Patricia changed expression, looked Judy in the eyes with a nuanced smile, nodded imperceptibly. Judy then asked her how she was feeling at that precise moment, and the patient replied that she felt ‘on the edge,” a state that—with Judy’s help—Patricia understood as of alarming uncertainty about the safety of the relationship. Judy then helped Patricia to recognize that her hypervigilant and mistrustful part protected her from her need for closeness, and then said to her: “it seems that when you seek my closeness and you obtain it, you initially feel comfort but, immediately afterwards, you fear that the connection with me is unreal and that I have the power to hurt you, and consequently this makes me very dangerous at the very moment in which I seem closest to you.” Patricia nodded sadly.

This session marked a turning point. During the following weeks, breaks in the therapeutic relationship occurred on several occasions: Patricia suddenly became distrustful and withdrawn; but Judy promptly repaired these ruptures by helping Patricia to reflect on the fact that it was the fear that Judy was not really interested in her that generated the distrust and closure. In one session during the sixth month of therapy, Patricia was able to share with the therapist a traumatic scenario from her childhood: her parents who harshly scolded her when she cried as a child.

## 5. Outcome

After 10 months of therapy, Patricia’s persecutory delusions essentially disappeared. She was able to return to university classes. Occasionally, her relational solicitations generated alarm and rumination about the possible malevolent intentions of others, but Patricia was able to interactively regulate this state of suffering with the therapist, for example by texting her, or describing her internal state in a diary, imaging she was talking with the therapist. Patricia became more and more able to see her contingent persecutory ideas as fantasies not necessarily mirroring reality, and to sooth her suffering. Patricia’s awareness of her own and others’ minds and awareness of herself as someone capable of mastering psychological and interpersonal challenges improved. In various situations she began to feel a sense of self-efficacy and agency. Her social withdrawal did not diminish significantly, but she seemed progressively to acquire the ability to enjoy moments of well-being alone through contact with nature, and to resort to the active search for these moments also to regulate and prevent states of suffering.

## 6. Discussion

In the last few decades, approaches focusing on the dysfunctions in metacognition are enriching the landscape of the psychological treatment of psychosis. Furthermore, some of the authors who follow a metacognitive-oriented approach to psychosis, emphasize the need to give relevance within them to countertransference and intersubjective processes. Taking this theoretical line, in this paper we focus on the impact of the therapist’s developmental trauma on the therapeutic relationship. We make a “parallel” analysis of the clinical case of a young woman with symptoms of psychosis, and the supervision process of the case. It is shown that the therapeutic relationship can be significantly conditioned by the therapist’s developmental history; and how a process of supervision particularly focused on the exploration of the traumatic elements of this history can effectively improve the therapist’s metacognitive abilities (both the ability to understand one’s own mind and that of the patient), and promote a functional patient-therapist intersubjective attunement and a good clinical outcome. Of note, the therapist presented in this case had followed a personal psychotherapy, during which she had had the possibility of elaborating the traumatic elements of her developmental history; but she never realized how significant a role such elements played in reducing her ability to attune to her patients, nor the negative impact that the re-emergence of her DT in the therapeutic relationship had on her metacognitive abilities. This sheds light on the need to consider such a process of supervision complementary to personal psychotherapy for therapists engaged in the treatment of patients with psychosis.

Our paper has a series of limitations. The first is that the successful outcome of the clinical process may be attributable to factors external to supervision, such as the short duration of illness, the patient’s young age, and the patient’s drug therapy. In addition, it seems that the apparent positive effect of supervision on the clinical outcome is mediated by the link that the supervision establishes between therapist’s personal history and therapeutic practice; but the effect produced by this link may have been relevant only because of its character of novelty for the therapist described in our paper, with her personal developmental history and her specific training background. This character of novelty could have been less prominent with a psychodynamic/psychoanalytic therapist, since in this theoretical-clinical context therapists are regularly encouraged—as part of their training—to reflect on their emotional responses with their patients and how their own internal dynamics may impact the therapeutic relationship.

Finally, our theoretical-clinical speculation opens up several lines of research. One of these could be the exploration of the correlation between the patient’s reduction of symptoms and emotional dysregulation, on the one hand, and, on the other hand, the subjective perception of being understood and internal soothing that the therapist experiences in the context of the supervisory relationship. Another interesting line of research could be the exploration of the correlation between therapist’s metacognition and capacity to intersubjectively attune with a patient with psychosis.

## 7. Patient perspective

While we write this paper, Patricia’s therapy is ongoing, with a session every 2 weeks; the therapeutic relationship is very solid. Patricia has started to go out sometimes with a university colleague who shares her passion for nature. In one of the last sessions of this phase, the therapist received explicitly positive feedback about the treatment from the patient.

## Data availability statement

The raw data supporting the conclusions of this article will be made available by the authors, without undue reservation.

## Ethics statement

Ethical review and approval were not required for the study on human participants in accordance with the local legislation and institutional requirements. The patients/participants provided their written informed consent to participate in this study. Written informed consent was obtained from the participant/patient(s) for the publication of this case report.

## Author contributions

GS was responsible for conceptual work, supervision, and drafting the first version of the manuscript. TS contributed to the case conceptualization and conceptualization of the supervisory process. LB, MC, NS, GF, NM, RM, AP, MP, and AS contributed to conceptual work, manuscript revision, and linguistic review. All authors contributed to the article and approved the submitted version.

## Funding

The work was supported by the Published with a contribution from 5 × 1000 IRPEF funds in favor of the University of Foggia, in memory of Gianluca Montel.

## Conflict of interest

The authors declare that the research was conducted in the absence of any commercial or financial relationships that could be construed as a potential conflict of interest.

## Publisher’s note

All claims expressed in this article are solely those of the authors and do not necessarily represent those of their affiliated organizations, or those of the publisher, the editors and the reviewers. Any product that may be evaluated in this article, or claim that may be made by its manufacturer, is not guaranteed or endorsed by the publisher.

## Footnotes

^1^Even if partially overlapping, the concepts of mentalization and metacognition present a major difference. Metacognition is a conceptual construct framed in a constructivist-oriented background; it consists of a spectrum of mental activities that involve thinking about thinking. It refers to mental activities ranging from discrete acts in which people recognize specific thoughts and feelings to more synthetic acts in which an array of intentions, thoughts, feelings, and connections between events are integrated into a larger complex representation; metacognition also includes “mastery,” namely the ability to use metacognitive knowledge to solve the psychologically or emotionally challenging events and social problems occurring in daily life (10). The construct of mentalization is framed in a psychoanalytic background and, in particular, in the development of psychodynamic approaches for adults diagnosed with borderline personality disorder. According to this background, mentalization would develop in the context of secure attachment from the early phases of interaction with caregivers (8). Since the authors are engaged in the field of metacognition, in this paper this construct will be preferred.

^2^Both the name of the patient and therapist are fictitious.

^3^For further information about a similar clinical conceptualization, based on the metaphor of a dialectic among identity parts, see (33, 34).
